# Metabolic syndrome and life style factors among diabetes patients attending in a teaching hospital, Chitwan

**DOI:** 10.1371/journal.pone.0286139

**Published:** 2023-05-25

**Authors:** Kalpana Sharma, Sunita Poudyal, Hem K. Subba, Saurav Khatiwada

**Affiliations:** 1 School of Nursing, Chitwan Medical College, Bharatpur, Nepal; 2 Departments of Endocrine Medicine, Chitwan Medical College, Bharatpur, Nepal; University of Botswana School of Medicine, BOTSWANA

## Abstract

**Background:**

Metabolic syndrome (MetS) is associated with an increased incidence of chronic complications and mortality of diabetes patients. Prevention and treatment of MetS is important means of lowering the risk of cardiovascular diseases and mortality.

**Objective:**

This study aimed to find out metabolic syndrome and life style factors among diabetes patients.

**Methods:**

A cross-sectional survey was carried out among 296 patients with type 2 diabetes mellitus attending Chitwan Medical College Teaching Hospital. Consecutive sampling technique was used to select sample. Data were collected from 15^th^ December 2021 to 15^th^ March, 2022 using Interview Schedule, bio-physiological measurement and record review. Obtained data were analysed in SPSS version 20 for window using descriptive and inferential statistics. Chi-square test was applied to measure the association between the variables. Logistic regression analysis was performed to identify the factors associated with metabolic syndrome.

**Result:**

Findings revealed that the prevalence of MetS was 66.2% and 58.4% in patients according to International Diabetes Federation (IDF) and National Cholesterol Education Program Adult Treatment Panel III (NCEP ATP III) criteria respectively. The most common MetS parameters were raised fasting plasma glucose (94.6%) and abnormal waist circumference (78.4% in IDF criteria) while the least prevalent parameter was reduced HDL level (43.2%). Majorities of the patients were non-vegetarian (85.5%), had poor dietary compliance (poor-46.3%, very poor-32.1%), overweight/obese (65.5%), and suffered from moderate stress (90.1%). Bivariate analysis showed that MetS as per NCEP ATP criteria was significantly associated with gender (p = 0.006), occupation (p = 0.007), presence of other co-morbid condition (<0.001) and sleep problem (p = <0.001). However, MetS as per IDF criteria was significantly associated with age (p = <0.028), duration of diabetes (p = <0.001), follow-up visit (p = <0.030), blood sugar monitoring (p = <0.009) and physical activity of diabetes patients (p = <0.001). Further logistic regression analysis revealed that sleep problem (AOR = 21.812;95%CI = 8.512,55.894) and presence of other comorbidities (AOR = 4.024;95%CI = 2.220,7.295) were the significant factors of metabolic syndrome.

**Conclusion and recommendation:**

Metabolic syndrome is high in patients with type 2 diabetes mellitus. Therefore, treating physicians and other health workers need to monitor MetS parameters regularly to reduce the risk of cardiovascular diseases, stroke and premature death.

## Introduction

Metabolic Syndrome (MetS) is one of the cluster of cardio-metabolic dysfunctions which is characterized by increase in fasting blood sugar (FBS), abdominal circumference (AC), arterial pressure (AP), triglycerides (TG), and reduction in high-density lipoprotein cholesterol (HDL) [1]. Different criteria used for diagnosing MetS provide differing results. According to National Cholesterol Education Program Adult Treatment Panel III (NCEP ATP III) definition, MetS is present if three or more of the following components met: abdominal obesity, raised TG, low HDL, hypertension, and elevated fasting glucose. International Diabetes Federation (IDF) 2005 focus on the presence of central obesity, together with 2 or more other components [2].

People with Diabetes Mellitus (DM) have greater risk of developing MetS compared to general people [3]. MetS also increases the risk of cardiovascular diseases (CVD), and premature death as well as increase the cost related to health care [4]. Many studies reported the common prevalence of MetS among type 2 diabetes mellitus patients [4–7]. Research evidence showed the significant association between metabolic syndrome and other socio-demographic and life-style factors such as age, sex, dietary patterns, physical activity, overweight and obesity [5]. Patients suffering from DM with MetS are at an increased risk of developing chronic diabetes complications [8], and decrease survival rate by at least 10 years [9]. Therefore, when treating at-risk patients, physicians and other health care workers need to pay special attention to cardiovascular risk factors to reduce morbidity and mortality. There is gap in literatures regarding metabolic syndrome and associated factors in Nepalese context especially in the Chitwan district. Hence, this study aimed to find out metabolic syndrome and life style factors among diabetes patients attending in a Teaching Hospital which will be helpful to emphasize the DM patients need, particularly careful follow-up and active intervention for cardiovascular risk factors.

## Materials and methods

### Study design, period and setting

A cross-sectional study was carried out in Chitwan Medical College-Teaching Hospital (CMC-TH), Bharatpur-10, Chitwan from November 2021 to May 2022. This hospital is 700 beded tertiary care hospital, located in the central Nepal where highly qualified health man powers are available and patients from different parts of the country visit for the treatment. This hospital provides general out-patient, inpatients, specialist and super-specialty services. Medical department have general as well as super-specialties services in endocrinology, cardiology, gastroenterology, respiratory and nephrology. Medicine and endocrine OPD provides services on all working days (Sunday to Friday) from 8am to 4pm. On an average 14–15 diabetes patients attend per day and total number of patients attended in OPD during 3 months period was 1350.

### Study population and eligibility criteria

Those patients who were clinically diagnosed to have diabetes mellitus and attending at CMC-TH during data collection period were the study population. This study included patients who were: (i) 20 years and above (ii) clinically diagnosed to have Type 2 diabetic mellitus through the criteria of American Diabetic Association (2021) (Fasting blood sample ≥126 mg/dl nil per oral for at least 8 hours, post prandial ≥ 200 mg/dl) for at least 1 year (iii) treated with anti-diabetic medications and (v) willing to participate in the study. This study excluded patients who (i) had history of other co-existing serious illness or inflammatory disorders such as heart failure, myocardial infarction, chronic kidney disease, (ii) were pregnant or lactating their baby (iii) were on prolong steroid therapy and lipid lowering drugs, and (iv) active urinary tract infection through routine urine examination.

### Sample size determination and sampling technique

Cochrane’s formula (n_0_ = $\frac{z^{2}pq}{d^{2}}$) was used to calculate the sample size needed for the study at a 95% confidence interval (z = 1.96) and 5% allowable error (d = 0.05). Assuming a p of 0.74 (74% prevalence of metabolic syndrome among type II diabetic patients in Nepal) [10], the minimum required calculated sample size was 296. There were 1350 patients (N) visited OPD during 3 months period. Using sample size calculation formula for finite population, calculated sample size was 243. By adding 20% non-response rate, final sample size (n) was 292≈296.

Non-probability consecutive sampling technique was used for recruiting the desired sample size. Those patients who met the study criteria and came to the selected OPD during data collection period were taken as study sample and data were collected till the desired sample achieved.

### Operational definitions

#### Perceived stress

It referred to emotional feelings or thoughts that an individual has about how much stress they are under at a given point in time or over a given time period. It was measured by using perceived stress scale (PSS) [11] containing 10 items. Each item was rated to 0 to 4 score. Total perceived stress score was calculated and further classified as: Low: (0–13), moderate: (14–26), high: (27–40).

#### Physical activity

It referred to any bodily movement produced by skeletal muscles that increase energy expenditure above a basal level. It was measured by physical activity questionnaire-short form [12]. It was further classified into low (<600 METs), moderate (600–1499.99 METs) and high (≥1500 METs) activities.

#### Metabolic syndrome

It was defined according to Adult Treatment Panel III (ATP III) and International Diabetes Federation (IDF) criteria respectively [13]. A person was diagnosed with metabolic syndrome if he/she had any three of the following modified NCEP ATP III criteria:

Central obesity: waist circumference ≥ 102 (male), ≥ 88 cm (female)
Dyslipidemia: TG ≥1.7 mmol/L (150 mg/dl)
Dyslipidemia: HDL-C< 40 mg/dL (male), < 50 mg/ dL (female)
Blood pressure ≥ 130/85 mmHg
Fasting plasma glucose ≥ 5.6 mmmol/l (100 mg/dl)

According to the International Diabetes Federation consensus worldwide (2005), person was diagnosed with metabolic syndrome if he/she had central obesity (defined as waist circumference with ethnicity specific values for South Asian descent i.e. a waist circumference of ≥90 cm for men and ≥80cm for women) and any two of the following criteria:

Raises triglycerides: >150 mg/dl (1.7 mmol/L), or specific treatment for this lipid abnormality.
Reduced HDL cholesterol level <40mg /dl (1.03 mmol/L) in males, <50 mg/dl (1.29mmol/L) in female or specific treatment for this lipid abnormalities
Raised blood pressure: Systolic BP>130 mmHg or diastolic BP >85mm Hg or treatment of previously diagnosed hypertension
Raised fasting plasma glucose: >100 mg/dl (5.6 mmol/L) or previously diagnosed type 2 diabetes

An earlier study conducted in Nepal [10] revealed that an NCEP ATP III classification is preferable to IDF standards for diagnosing MetS cases among diabetic patients in Nepal. So, in regression analysis, the ATPIII specification was applied.

#### Monthly family income

It was classified into three categories as inadequate (if family income unable to fulfil their basic needs for a month), adequate (if family income was able to fulfilled basic needs) and surplus (if there was extra saving after fulfilling basic needs).

#### Smoking status

Patients were classified as never smoker (who had never smoked, or smoked less than 100 cigarettes in his or her lifetime), ex-smoker (who smoked at least 100 cigarettes in his or her lifetime but has not smoked in the last 28 days) and current smoker (who smoked >100 cigarettes in his or her lifetime and has smoked in the last 28 days)

#### Alcohol habit

Participants were categorized as lifetime abstainer (never consumed alcohol in their lifetime), former drinker (used to drink alcohol but have abstained for the last 12 months), current drinker (consumed alcohol at least once during the last 12 months and who currently drink).

#### Follow up visit

Patients were classified as regular follow-up visit group if they visited the physician according to their prescription and irregular group if they did not visit physician as schedule.

#### Blood glucose monitoring

Participants were classified as regular blood monitoring group if they monitored their blood glucose according to physician prescription and irregular if they did not follow the schedule.

#### Dietary compliance

It referred to following of dietary suggestions of physician/endocrinologist for the management of diabetes mellitus. It included foods to avoid, number of meals, carbohydrate amount, food variety and sugar. Each item was rated 0 to 1 where 0-complaince and 1-non-complaince.

**Table:** 

Items	Compliance (0)	Non-compliance (1)
Food to avoid	Not included	Included
Number of meals	≥ 4	<4
Carbohydrate amount	Less	More
Food variety	More	Less
Sugar	No	Yes

Dietary compliance total score was calculated by summing all the items score where 5-very bad, 4-bad, 3-neutral, 2-good, 1-very good.

#### Sleep problem

Patients who slept 7–8 hours per day without difficulty in initiating and maintaining sleep are grouped as adequate sleep group whereas those patients who could not sleep for 7 hours per day and had difficulty in initiating and maintaining sleep were grouped as sleep problem group.

### Data collection tools and measurement

Three types of research instruments were used in the study. Structured interview schedule was used for recording socio-demographic information, disease and treatment related variables and lifestyle factors of the respondents. Bio-physiological measurements were performed for the metabolic parameters (height, weight, waist circumference, blood pressure, blood sugar and blood cholesterol). Record review was done for medications, history of other illness and findings of other investigations. Perceive Stress Scale [11] and physical activity questionnaire-short form [12] were used to measure stress and physical activity of respondents.

Patients who met the study criteria were identified, asked them for the participation in the study, and objective was explained. Those patients who agreed to participate and be ready to remain at least 12–14 hours fasting to give blood sample were recruited for study. Patients’ socio-demographic, clinical and lifestyle factors were collected from direct interview method and medical records. Sleep was evaluated on the basis of sleep duration (7hour to 8 hour), difficulty in initiating and maintaining sleep. Anthropometric measurement (height in meter, weight in kg, waist and hip circumferences in centimetre, blood pressure in mmHg) was recorded. Patients were instructed to wear light clothing and no shoes during physical measurements. Weight and height were measured using portable weighing scale (Krups, India) and stadiometer. Measurement of waist circumference was taken at the narrowest indentation midway between the lowest rib and the iliac- crest on the bare skin at the end of normal gentle respiration. Blood pressure was measured twice in 10 minutes apart by using mercury sphygmomanometer (model BK1005) following recommendations from 2020 International Society of Hypertension Global Hypertensive Practice Guideline. Then the mean reading was calculated. After measuring height in meter and weight in kilogram, body mass index was calculated using formula (weight in kg÷ height in m^2^).

Next day, patients came to OPD to give their blood sample. Laboratory technician collected the blood sample and used syringes were kept in puncture proof container. Then, sample was sent to lab within 10 minutes for the analysis. Sample for lipid profile and glucose were centrifuged in 5000 rpm for 5 minutes and serum was separated. Then it was kept in analyser of fully automated dimension RXL Max Siemens machine to determine fasting blood sugar, triglycerides and HDL. High Performance Liquid Chromatography (HPLC) method (H9, Lifotronic) was applied for the determination of HbA1c. Test was completed within 2 hours from sample collection. Blood samples were discarded after the biochemical measurements. The data were collected by researchers themselves from 15^th^ December 2021 to 15^th^ March 2022.

#### Data quality control

The questionnaire were translated into Nepali language and back translated to English language by language expert for the consistency. The questionnaire were pre-tested among 30 type 2 diabetes patients (10% of sample) attending endocrine OPD of CMC-TH but they were excluded for the final study. Nepali version questionnaire were used for the data collection. Anthropometric and laboratory measurement were taken using standard protocol. Each anthropometric parameter were taken twice and the measurement were repeated if the difference existed. Two hours briefing session was arranged on the data collection process for the data collectors and lab technician. Data were collected by researchers themselves. Biochemical assessment was completed by lab technician within 2 hours from sample collection.

#### Data management and analysis

Collected data were entered into Epi data 3.1 and exported to IBM SPSS (Statistical Package for Social Sciences) version 20.0 for window. Descriptive statistics were applied to report outcome variables and categorical variables. Chi-square test was performed to measure the association between potential factors and MetS. Variables with p-value <0.1 were transferred to a multivariable binary logistic regression model. Hosmer and Lemeshow goodness of fit test was applied to assess model fitness. Adjusted Odd ratio (AOR) with 95% confidence interval (CI) was reported to show the strength of association. Statistical significance was set at p value <0.05.

### Ethical consideration

Before data collection, ethical clearance was obtained from Nepal Health Research Council Ethical Review Board (Ref no 1426). Data collection permission was obtained from the Chitwan Medical College Institutional Review Committee (CMC-IRC) and hospital administration of CMC-TH. Participants were informed about the purpose of the study and the benefits they get from this study i.e. their laboratory test were done free of costs and reports were given to them. The written informed consent was obtained from all participants to maintain their right to self-determination. For the anonymity and confidentiality of information, code number was given to each participant instead of name, and they were assured that their information will not be disclosed to other unauthorized persons. Individual counselling was given based on their result. Patients who were diagnosed to have metabolic syndrome were referred to cardiologists for the further treatment. After analysis of data, all information containing forms were discarded and burned.

## Results

Majority of the diabetes patients were middle aged group (61.5%), belonged to Brahmin and Chhetri ethnicity (66.5%), lived in joint family (70.3%), and literate (79.7%). Highest percentages of patients were involved in household work (35.8%) and in agriculture (20.6%). Nearly two third (63.8%) of the patients suffered from diabetes for 5 and more years, 77.7% took oral hypoglycaemic agents and 54.7% had other co-morbid conditions (Table 1) and most common condition was hypertension (not shown in table). One hundred and three (34.8%) and 193 (41.9%) patients had taken lipid lowering drugs and anti-hypertensive medicine (Table 1).

**Table 1:** Socio-demographic and disease related characteristics of patients n = 296.

Variables	Number	Percent
**Age groups in years**		
Young (20–39 years)	19	6.4
Middle (40–59 years)	182	61.5
Elderly (>60 years)	95	32.1
**Gender**		
Male	145	49.0
Female	151	51.0
**Ethnicity**		
Bramin	138	46.6
Chhetri	59	19.9
Janajati	77	26.0
Dalit and Others	22	7.5
**Family type**		
Nuclear	88	29.7
Joint	208	70.3
**Marital status**		
Married	291	98.3
Unmarried	5	1.7
**Educational Status**		
Illiterate	60	20.3
Literate	236	79.7
**Occupation**		
Household work	106	35.8
Agriculture	61	20.6
Business	55	18.6
Retired	27	9.1
Service	22	7.4
Others	25	8.4
**Family income per months**		
Inadequate	2	0.7
Adequate	184	62.2
Surplus	110	37.2
**Duration of disease diagnosis in year**		
<5	107	36.1
5–10	112	37.8
>10	77	26.0
**Type of anti-diabetic medication**		
Oral	230	77.7
Injection	15	5.1
Mixed	51	17.2
**History of other comorbid-condition**		
Yes	162	54.7
No	134	45.3
**Taking lipid lowering treatment**		
Yes	103	34.8
No	193	65.2
**Taking treatment for hypertension**		
Yes	124	41.9
No	172	58.1
**History of hospital admissions in the last 1 months**		
No	274	92.6
Yes	22	7.4

Mean age (± SD) = 54.71(±10.52) Min age: 31.0 year Max age: 83.0 year

Median duration of DM (interquartile range):6 year (3 year-11 year)

Most of the patients were non-vegeterian (85.5%), more than three fourth (78.4%) had poor/very poor dietary compliance, nearly two third (65.5%) were overweight/obese and 53.7% involved in moderate/high level of physical activity. Still few percentage of patients were current smoker (8.1%) and current alcohol users (21.6%) and sleep problem (30.7%). On perceived stress, most of the patients (90.9%) had moderate level of stress whereas only 1.0% had high level of stress. Majority of patients went for regular follow-up visit (80.1%), and performed regular glucose monitoring (80.4%) (Table 2).

**Table 2:** Life style factors among patients n = 296.

Factors	Number	Percent
**Food Habits**		
Vegetarian	43	14.5
Non vegetarian	253	85.5
**Dietary Compliance**		
Very good	2	0.7
Good	17	5.7
Neutral	45	15.2
Poor	137	46.3
Very poor	95	32.1
**Smoking Habit**		
Never smoker	201	67.9
Past smoker	71	24.0
Current smoker	24	8.1
**Alcohol habit**		
Abstainer	177	59.8
Former drinker	55	18.6
Current drinker	64	21.6
**Other nicotinic substance use**		
No	249	84.1
Yes	47	15.9
**BMI**		
Underweight (<18.5)	7	2.4
Normal (18.5–24.99)	95	32.1
Overweight (25.0–29.9)	130	43.9
Obesity (30 and above)	64	21.6
**Physical Activity**		
No activity	116	39.2
Low	21	7.1
Moderate	119	40.2
High	40	13.5
**Perceived Stress Scale**		
Low (0–13)	24	8.1
Moderate (14–26)	269	90.9
High (27–40)	3	1.0
**Sleep problem**		
Present	91	30.7
Absent	205	69.3
**Regular follow-up**		
Yes	237	80.1
No	59	19.9
**Regular blood sugar monitoring**		
Yes	238	80.4
No	58	19.6

On examining each criteria of NCEP ATP III and IDF criteria separately for all patients, findings show that 78.4% of the patients had met the IDF criteria for central obesity or abnormal waist circumference whereas only 40.5% patients in NCEP ATP III criteria. Most of the patients (94.5%) had met the criteria for raised fasting plasma glucose, 57.8% had met the criteria for raised blood pressure, 52.4% for raised triglyceride and 43.2% for reduced HDL on both NCEP ATP III and IDF criteria (Table 3).

**Table 3:** Metabolic syndrome parameters among patients n = 296.

Metabolic Syndrome Parameters	Total (296) No. (%)	Male (145) No. (%)	Female (151) No. (%)
Central obesity or abnormal waist circumference (WC ≥90 cm for men and ≥80cm for women according to IDF Criteria)	231 (78.4)	112 (77.2)	120 (78.5)
Central obesity or abnormal waist circumference (WC ≥ 102 for male, ≥ 88 cm for female according to NCEP/ATP III criteria)	120 (40.5)	16 (11.0)	104 (68.9)
Raised blood pressure (130/85 mmHg)	171 (57.8)	83 (57.2)	88 (58.3)
Raised triglyceride level (≥150 mg/dl)	155 (52.4)	74 (51.0)	81 (53.6)
Reduced HDL (male < 40 mg/dl, female < 50 mg/ dL)	128 (43.2)	60 (41.4)	68 (45.0)
Raised fasting plasma glucose (≥ 100 mg/dl)	280 (94.6)	137 (94.5)	143 (94.7)

Out of 296 patients with diabetes mellitus, 66.2% and 58.4% of patients respectively had met the IDF and NCEP ATP III criteria for metabolic syndrome (Table 4).

**Table 4:** Prevalence of metabolic syndrome among patients n = 296.

Metabolic Syndrome	Total (n = 296) No.(%)	Male (n = 145) No.(%)	Female (n = 151) No.(%)
**According to IDF criteria**			
Presence	196 (66.2)	97 (66.9)	99 (65.6)
Absent	100 (33.8)	48 (33.1)	52 (34.4)
**According to NCEP ATP III critera**			
Presence	173 (58.4)	59 (40.7)	114 (75.5)
Absent	123 (41.6)	86 (59.3)	37 (24.5)

Sex, occupations and presence of other co-morbid conditions were significantly associated with the metabolic syndrome according to NCAP/ATP III criteria whereas gender and duration of disease diagnosis were significantly associated with metabolic syndrome according to IDF critera. However, none of other variables were significantly associated with the metabolic syndrome in the diabetes patients (Table 5).

**Table 5:** Association between metabolic syndrome and selected socio-demographic and disease related characteristics among patients n = 296.

Variables	Metabolic Syndrome NCEP/ATP Criteria					Metabolic Syndrome IDF Criteria		
	Absent No. (%)		Present No. (%)		p value	Absent No. (%)	Present No. (%)	p value
**Age groups in years**								
Young	8 (42.1)		11 (57.9)		0.821	8 (42.2)	11 (57.9)	**0.028**
Middle	78 (42.9)		104 (57.1)			70 (38.5)	112 (61.5)	
Elderly	37 (38.9)		58 (61.1)			22 (23.2)	73 (76.8)	
**Gender**								
Male	72 (49.7)		73 (50.3)		**0.006**	48 (33.1)	97 (66.9)	0.808
Female	51 (33.8)		100 (66.2)			52 (34.4)	99 (65.6)	
**Ethnicity**								
Bramin/chhetri	78 (39.6)		119 (60.4)		0.932	67 (34.0)	130 (66.0)	0.937
Janajati	35 (45.5)		42 (54.5)			25 (32.5)	52 (67.5)	
Dalit and others	10 (45.5)		12 (54.5)			8 (36.4)	14 (63.6)	
**Family type**								
Nuclear	34 (38.6)		54 (61.4)		0.508	30 (34.1)	58 (65.9)	0.942
Joint	89 (42.8)		119 (57.2)			70 (33.7)	138 (66.3)	
**Education**								
Illiterate	20 (33.3)		40 (66.7)		0.148	22 (36.7)	38 (63.3)	0.597
Literate	103 (43.6)		133 (56.4)			78 (33.1)	158 (66.9)	
**Occupation**								
Service	10 (45.5)		12 (54.5)		**0.007**	7 (31.8)	15 (68.2)	0.505
Business	34 (61.8)		21 (38.2)			20 (36.4)	35 (63.6)	
Agriculture /household work	60 (35.9)		107 (64.1)			60 (35.9)	107 (64.1)	
Others	19 (36.5)		33 (63.5)			13 (25.0)	39 (75.0)	
**Family income per months**								
Surplus	52 (47.3)		58 (52.7)		0.292	34 (30.9)	76 (69.1)	0.299
Adequate	70 (38.0)		114 (62.0)			66 (35.9)	118 (64.1)	
Inadequate	1 (50.0)		1 (50.0)			0 (0)	2 (100.0)	
**Duration of disease diagnosis in years**								
<5	46 (43.0)	61(57.0)		0.682		74 (69.2)	33 (30.8)	**<0.001**
5–10	43 (38.4)	69 (61.6)				26 (23.2)	86 (76.8)	
>10	34 (44.2)	43 (55.8)				0 (0)	77 (100)	
**Type of anti-diabetic medication**								
Oral	102 (44.3)		128 (55.7)	0.109		83 (36.1)	147 (63.9)	0.122
Injection	3 (20.0)		12 (80.0)			6 (40.0)	9 (60.0)	
Mixed	18 (35.3)		33 (64.7)			11 (21.6)	40 (78.4)	
**History of hospital admission**								
No	7 (31.8)		15 (68.2)	0.335		10 (45.5)	12 (54.5)	0.229
Yes	116 (42.3)		158 (57.7)			90 (32.8)	184 (67.2)	
**History of other comorbid-condition**								
Yes	45 (28.0)		116 (72.0)	**<0.001**		49(30.4)	112 (69.6)	0.183
No	78 (57.8)		57 (42.2)			51(37.8)	84 (62.2)	
**Taking lipid lowering treatment**								
No	80 (41.5)		113 (58.5)	0.961		70(36.3)	123(63.7)	0.216
Yes	43 (41.7)		60 (58.3)			30(29.1)	73 (70.9)	
**Taking treatment for hypertension**								
Yes	67 (39.0)		105 (61.0)	0.285		58(33.7)	114(66.3)	0.979
No	56 (45.2)		68 (54.8)			42(33.9)	82 (66.1)	

There was statistically significant association between metabolic syndrome with physical activity (p = <0.001), regular follow up visit and regular blood glucose monitoring of the diabetes patients according to IDF criteria. Similarly, patients with DM who had sleep problem had significantly higher metabolic syndrome compared to patients who did not have sleep problem (p = <0.001) according to NCEP ATP III criteria. But metabolic syndrome was not significantly associated with other life style factors like food habits, dietary compliance, smoking habit, BMI etc (Table 6).

**Table 6:** Association between metabolic syndrome according to ATP III Criteria and selected life style factors among patients n = 296.

Variables	Metabolic syndrome NCEP ATP III			Metabolic syndrome IDF		
	Absent No. (%)	Present No. (%)	p value	Absent No. (%)	Present No.(%)	P value
**Food habits**						
Vegetarian	14 (32.6)	29 (67.4)	0.195	12(27.9)	31 (72.1)	0.378
Non vegetarian	109 (43.1)	144 (56.9)		88 (34.8)	165 (65.2)	
**Dietary Compliance**						
Very good/good	97 (41.8)	135 (58.2)	0.910	81 (34.9)	151 (65.1)	0.687
Neutral	19 (42.2)	26 (57.8)		14 (31.1)	31 (68.9)	
Poor	7 (36.8)	12 (63.2)		5 (26.3)	14 (73.7)	
**Smoking Habit**						
Never	81 (40.3)	120 (59.7)	0.089	76 (37.8)	125 (66.2)	0.065
Ex-smoker	27 (30.0)	44 (620)		16 (22.5)	55 (77.5)	
Current smoker	15 (62.5)	9 (37.5)		8 (33.3)	16 (66.7)	
**Alcohol habit**						
Current	34 (53.1)	30 (46.9)	0**.046**	24 (37.5)	40 (62.5)	0.113
Former	17 (30.9)	38 (69.1)		12 (21.8)	43 (78.2)	
Abstainer	72 (40.7)	105 (59.3)		64 (36.2)	113 (63.8)	
**BMI**						
Underweight (<18.5)	1 (14.3)	6 (85.7)	0.334	4 (57.1)	3 (42.9)	0.056
Normal (18.5–24.99)	40 (42.1)	55 (57.9)		24 (25.3)	71 (74.7)	
Overweight /obesity (≥25)	82 (42.3)	112 (57.7)		72 (37.1)	122 (62.9)	
**Sleep problem**						
No	117 (57.1)	88 (42.9)	**<0.001**	75 (36.6)	130 (63.4)	0.126
Yes	6 (6.6)	85 (93.4)		25 (27.5)	66 (72.5)	
**Physical activity**						
Low	58 (42.3)	79 (57.7)	0.598	63 (46.0)	74 (54.0)	**<0.001**
Moderate	46 (38.7)	73 (61.3)		37 (31.1)	82 (68.9)	
High	19 (47.5)	21 (52.5)		0 (0.0)	40 (100.0)	
**Perceived stress**						
Low (0–13)	12 (50.0)	12 (50.0)	0.661	7 (29.2)	17 (70.8)	0.249
Moderate (14–26)	110 (40.9)	195 (59.1)		93 (34.6)	176 (65.4)	
High (27–40)	1 (33.3)	2 (66.7)		0 (0/0)	3 (100.0)	
**Regular follow-up visit**						
Yes	95 (40.1)	142 (59.9)	0.304	73 (30.8)	164 (69.2)	**0.030**
No	28 (47.5)	31 (52.5)		27 (45.8)	32 (54.2)	
**Regular blood sugar monitoring**						
Yes	95 (39.9)	143 (60.1)	0.247	72 (30.3)	166 (69.7)	**0.009**
No	28 (48.3)	30 (51.7)		28 (48.3)	30 (51.7)	

A logistic regression was performed to ascertain the factors associated with metabolic syndrome. This model explained 43.1% (Nagelkerke R^2^: 0.431) of the variance in the prevalence of metabolic syndrome among diabetes patients. Patients who had sleep problems and had comorbidity were 21.812 and 4.024 times more likelihood to develop metabolic syndrome compared to patients who did not have sleep problem and had no other comorbidity. It indicates that sleep problem and presence of comorbid conditions were the significant predictor of metabolic syndrome among patients with diabetes mellitus. However, gender, occupation, alcohol habit and smoking habit were not the significant predictor of metabolic syndrome (Table 7).

**Table 7:** Logistic regression model on factors of metabolic syndrome according to NCEP/ ATP III criteria among patients.

	Unstandardized *B*	p	Standardized *B*	p	95% CI for *B*
**Gender**					
Male	Ref				
Female	1.934	0.006	0.939	0.870	0.440–2.004
**Occupation**					
Business	Ref				
Service	0.356	0.010	0.751	0.557	0.289–1.953
Agriculture	0.691	0.479	1.273	0.700	0.374–4.338
Others	1.027	0.936	1.542	0.325	0.651–3.653
**Co-morbidity**					
No	Ref				
Yes	3.527	<0.001	4.024	<0.001	2.220–7.295
**Sleep problem**					
No	Ref				
Yes	18.835	<0.001	21.812	<0.001	8.512–55.894
**Alcohol habit**					
Current user	Ref				
Former user	2.533	0.016	2.093	0.115	0.835–5.247
Abstainer	1.653	0.087	0.925	0.848	0.415–2.062
**Smoking Habit**					
Never	2.469	0.042	3.533	0.390	1.063–11.747
Ex-smoker	2.716	0.040	2.272	0.218	0.616–8.375
Current smoker	Ref				

*Variable (s) entered on step 1*: *sex*, *occupation*, *comorbidity*, *sleep problem*, *histry of alcohol use*, *history of smoking*, *Dependent variable*: *prevalence of metabolic syndrome*, *Nagelkerke R*^*2*^: 0.431, Hosmer and Lemeshow test (x2-12.440, p = 0.133, df-8)

## Discussion

This study aimed to find out the metabolic syndrome and life style factors for the early diagnosis and timely treatment of MetS in patients with type 2 DM. In this study, prevalence of MetS was 66.2% and 58.4% respectively according to IDF and modified NCEP/ATP III criteria. This findings is similar to previous study from Nepal which reported 66.8% and 73.9% of metabolic syndrome using IDF and NCEP-ATP III, criteria respectively [10]. It indicates that metabolic syndrome is high among diabetes patients which is expected because they were suffering from type 2 diabetes which itself is an entity of the MetS.

Similar finding was also reported by the study conducted in India in which 77.2% prevalence of MetS among urban Indian diabetic patients as per NCEP/ ATPIII [14]. Likewise, Imam and colleagues [15] revealed 79.7.2% and 68.1% metabolic syndrome among diabetic patients in Karachi as per NCEP/ ATPIII and IDF criteria, Foroozanfar et al. [16] showed 73.4% & 64.9% prevalent of metabolic syndrome among diabetes patients in Iran, and Zerga and colleagues [5] revealed 50.3% and 59.4% metabolic syndrome in Ethiopia according to 2005 International Diabetes Federation and revised ATP III criteria respectively.

However, one study in Nepal [17] found slightly higher prevalence of metabolic syndrome in diabetes patients (71% and 82% respectively according to NCEP/ATP III and IDF criteria) compared to present study findings. Likewise, other studies from India [18], Ghana [19], and United Arab Emirates [20] reported lower rate of metabolic syndrome among diabetes patients according to NCEP-ATP III criteria. The discrepancy rates in prevalence of MetS between these studies could be due to differences in ethnic or geographical areas as well as approaches used to measure MetS of study participants.

On the metabolic syndrome parameters, central obesity or abnormal waist circumference was the highly prevalent parameter of metabolic syndrome in IDF criteria compared to modified NCEP ATP III criteria (78.4% in IDF criteria, 40.4% NCEP ATP III criteria). This difference is due to use of different cut-off points in the tool because IDF uses waist circumference with ethnicity specific values for South Asian descent (a waist circumference of ≥90 cm for men and ≥80cm for women) whereas modified NCEP ATP III criteria defined central obesity if waist circumference ≥ 102 for male, ≥ 88 cm for female. Excluding fasting blood glucose (94.6%), other metabolic parameters present in diabetes patients were hypertension (57.8%) followed by raised triglyceride (52.4%) and reduced HDL (43.2%) on both NCEP ATP III and IDF criteria. Similarly, a study conducted in other part of Nepal showed higher prevalence of metabolic parameters such as central obesity (IDF-99.9%, ATP-III-55.9%), low HDL (IDF-93.5%, ATPIII-95.2%), HTN (IDF-62.0%, ATPIII-72.1%), and raised TG (IDF-58.2%, ATPIII-76.8%) in diabetes patients [10].

Our findings are consistent with the result of the study done in Ethiopia [21] which showed obesity (59.7%) as the commonest metabolic parameters followed by triglyceride (45.1%), HTN (41.3%) and low HDL-c (34.4%) in DM patients. Another study in India [22] also showed central obesity and hypertension as the most frequently occurring risk factors in diabetes patients. Likewise, study in Ghana [23] reported high waist circumference among 60.9% of diabetes patients. The reasons behind this might be due to fact that some anti-diabetes medications (insulin and sulfonylureas and glitazones) promote weight gain and certain anti-hypertensives actually increase insulin resistance.

Our study revealed that, patients with higher duration of DM (>5 years) had greater risk of getting MetS according to IDF criteria. This finding is consistent with the studies conducted in Ghana [23] and Ethiopia [5, 21] where patients with more than 5 years disease duration have higher chance of having metabolic syndrome. Similar findings were also reported in other studies [24, 25]. It might be due to the fact that dietary and physical activity modifications are most common initial therapies in T2DM patients and those who have been treated for a long time may have poor compliance with the medications as well as other physical activities hence resulting in at greater risk of MetS. However, a prior study in Ethiopia found that there was no effect of treatment duration on MetS development which is similar with the finding of our study using NCEP ATP criteria [26].

In this study, prevalence of metabolic syndrome was higher among female (66.2%) compared to male (50.3%) diabetes patients as per NCEP ATP III criteria and the difference was statistically significant (p = 0.006). Other studies conducted in Nepal [17], India [14], Ethiopia [5] and Ghana [23] also showed higher prevalence of metabolic syndrome in women. This might be due to the fact that significant metabolic changes accompany women’s transition from the pre-menopause to the post-menopause state, and oestrogen insufficiency may raise their risk of developing MetS. In addition, women are mostly engaged in sedentary occupation and their involvement to physical activities is minimal. However, the prevalence of MetS was slightly higher in male (66.9%) compared to women (65.6%) diabetic patients as per IDF criteria but the difference was not statistically significant. Similarly, study finding from urban North-Central Nigeria revealed the higher metabolic syndrome among men compared to women [27]. The authors provided evidence to support their claim that women’s high activity levels may have contributed to men’s unusually higher prevalence of MetS.

With regard to age, our study showed that MetS was higher among elderly and age was significantly associated with MetS according to IDF criteria indicating that older people have higher chances to get metabolic syndrome. It is consistent with the findings of other studies [4, 5, 21] which reported the presence of MetS in older age. The possible reasons might be due to the facts that physical activity diminishes as the people get older and they become dependence on other. An overall increasing trend with advancing age might be contributed to an evolution of insulin resistance, hormonal alterations, and increase in visceral adipose tissue with age [28].

Our study revealed that just more than half of the diabetes patients engaged in the moderate to high level of physical activity whereas nearly half had sedentary lifestyle. Evidence have shown that physical activity improves glycaemic control and reduces the risk of cardiovascular disease (CVD) and mortality in patients with type 2 diabetes (T2D). Further, low level of physical activity such as walking has also beneficial effects on reducing the risk of T2D, CVD and mortality [29]. Studies from Ethiopia [5, 21] revealed that patients with sedentary behaviours had higher odds of metabolic syndrome. This may be due to the fact that obesity, insulin resistance, and impaired lipid metabolism are all caused by sedentarism. Contrary to existing evidence, present study showed that patients with high intensity physical activity had significantly higher metabolic syndrome compared to low physical activity. The possible reasons behind this might be due to the facts that patients started high physical activities only after onset of problems and also fear of worst possible outcomes.

This study showed that 21.6% of diabetes patients were taking alcohol currently and only 8.1% patients were current smokers. Smoking consumption was not significantly associated but intake of alcohol was significantly associated with metabolic syndrome. This is inconsistent with the study done in United Arab Emirates in which smoking was significantly associated with MetS among the Arab non-Emirati population [20].

In this study, most (90.9%) of the DM patients had moderate stress whereas very few (1.0%) had high stress according to perceived stress scale. Our finding showed higher stress than the finding of the studies conducted in India [30] and Ethiopia [5] which showed perceived stress among 39.0% and 23.2% of diabetes patients respectively. The difference in the findings obtained using the same instruments might be due to usage of different cut-off scores to define stress and different definition used to define MetS. Our study also showed that perceived stress was not the significant factors of metabolic syndrome. However, Kaur [31] revealed the significant association between stress predisposition and MetS. Similarly, study from Findland [32] discovered that stressful life experiences were linked to insulin resistance, obesity, and TGs and were considered to be bad indicators of metabolic health.

This study found sleep problem in nearly one third (30.7%) diabetes patients. This finding is in agreement with the observation of the studies conducted in India [28] and Ethiopia [5] where 47.77% and 48.2% of diabetes patients had sleep disturbance. This indicates that DM patients have sleep problem and that needs to be addressed promptly. In our study, odd of MetS among patients with sleep problem was 21.812 times more likely than patients without sleep problem. A similar finding was reported from India [31] in which sleep inadequacy was significantly associated with MetS. Persistent sleep insufficiency or deprivation raises the risk for diabetes and obesity and contributes to the aging process in addition to causing frequent mental and physical distress [33]. Similar to this, Coughlin et al. [34] have demonstrated that the prevalence of obstructive sleep apnea is independently correlated with an increase in the cardiovascular risk variables that make up the MetS.

This study also revealed that majority of patients were overweight and obese (65.5%), and poor/very poor dietary compliance (78.4%). But these factors were not found to be significantly associated with the metabolic syndrome. This finding is in line with the study done in Ethiopia [5]. Evidence reported that metabolically obese people of normal weight may be more susceptible to the MetS because of their abnormal body mass distribution and high fat mass of >30% [35]. In contrast, study from Ethiopia [5] reported higher categories of BMI as significant predictor of MetS. This may be the case when insulin resistance develops with increasing obesity. Also, overweight or obese people had more abdominal fat, which increases the risk of having high blood pressure and triglycerides.

In our study, educational status and economic status were not significantly associated with MetS. However, Birarra and colleague revealed that the patients who were of secondary school and above had significantly higher MetS. Further, author mentioned that higher level of education may indirectly lead to risky life style adoption in terms of dietary pattern and physical activity [4].

This study adds to the dearth of information regarding metabolic syndrome among diabetes patients attending a teaching hospital. Despite of this, it has some limitations (i)This is a cross-sectional study which fails to establish a causal relationship between MetS and its associated factors.(ii) As this study is conducted in single centre and OPD attended diabetes patients, the findings may not be easily generalized to all diabetic population of the country. (iii) Moreover, our study relied on self-reported life style factors such as diet, physical activity, smoking, alcohol habit and perceived stress which may have introduced bias on findings.

This study concluded that prevalence of metabolic syndrome was high among the patients with type 2 diabetes mellitus. Majority of the patients had high level of fasting blood sugar, central obesity/abnormal waist circumference, and raised blood pressure. Regarding lifestyle factors, moderate level of stress, poor dietary compliance, low physical activity, and overweight/obesity and sleep problem were common among the diabetes patients. Sleep problem and presence of other comorbidities were the identified factors of metabolic syndrome. So regular screening of the type 2 DM patients for the component of metabolic syndrome in the health care settings is essential to limit the risks of cardiovascular related morbidities and mortality. Further, counselling on regular follow-up and active intervention for cardiovascular risk factors is needed for the diabetes patients to promote their healthy lifestyle behaviours.

## Supporting information

S1 File The questionnaire used for the data collection. (DOCX)
