# Supplementary material for: Metabolic syndrome and life style factors among diabetes patients attending in a teaching hospital, Chitwan
Source: PLoS One. 2023 May 25;18(5):e0286139. doi: 10.1371/journal.pone.0286139 (PMC10212170; doi:10.1371/journal.pone.0286139)
Supplement: S1 File — (DOCX) [file pone.0286139.s001.docx]

**Metabolic Syndrome and Life Style Factors among Patients with Diabetes Attending Medicine OPD in Chitwan Medical College Teaching Hospital**

**Code Number: OPD Number**:

Date of Interview:

1. Name:
2. Age in year:
3. Sex:
4. Ethnicity:
5. Marital Status:
6. Type of family:
7. Education:
8. Occupation:
9. Family income per month:

9.1. Adequacy of monthly family income: (i) surplus (ii) adequate (iii) inadequate

1. Address:
2. Phone No:
3. Visitors Name and Number: …………………………………………………..
4. Duration of diabetes diagnosis in months/year: …………………………
5. Type of anti-diabetes medication used for the treatment**:** i. tablet ii. injection iii mixed

**Treatment Details**

| **SN** | **Medication Name** | **Dose** | **Duration** | **Remarks** |
| --- | --- | --- | --- | --- |
|  |  |  |  |  |
|  |  |  |  |  |
|  |  |  |  |  |
|  |  |  |  |  |
|  |  |  |  |  |
|  |  |  |  |  |
|  |  |  |  |  |

1. History of admission in the hospital due to illness within one month prior to this visit Yes/ No
2. Regular follow-up visit according to prescription: Yes/No
3. Blood sugar monitoring according to prescription: Yes/No
4. History of smoking: Never smoker/ex-smoker/current smoker

18.1 If smokers,

- 1. Age of initiation of smoking:
  2. Total year of smoking:
  3. Number of cigarette smoke per day…………

1. History of alcohol consumption: former users/current users/abstainer
   1. Frequency of alcohol consumption at least one alcoholic drink: Daily/5-6 days per week/1-4 days per

week/1-3 days per months/ less than once a month/

- 1. Amount of alcohol intake per day (in ml)…………………..

1. History of other substances use except smoking: Yes/No

If Yes, provide detail ……………………………

1. History of other co-morbid conditions: Yes/No

21.1 If yes, specify the conditions………………………………….

21.2 Spceify the types of drugs used for their illness

| **Name** | **Dose** | **Duration** |
| --- | --- | --- |
|  |  |  |
|  |  |  |
|  |  |  |
|  |  |  |

1. **Physical Activities Questionnaire**

|  | During the last 7 days, on how many days |  |
| --- | --- | --- |
| 22.1 | Did you do vigorous physical activities like heavy lifting, digging, aerobics, or fast bicycling? | _____ days per week  If No skip to question 3 |
| 22.2 | How much time did you usually spend doing vigorous physical activities on one of those days? | _____ hours per day  _____ minutes per day  ………..Don’t know/Not sure |
| 22.3 | Did you do moderate physical activities like carrying light loads, bicycling at a regular pace, or doubles tennis? Do not include walking | _____ days per week  ………..No skip to question 5 |
| 22.4 | How much time did you usually spend doing moderate physical activities on one of those days? | _____ hours per day  _____ minutes per day  …………..Don’t know/Not sure |
| 22.5 | Did you walk for at least 10 minutes at a time? | ____ days per week  ………..No, skip to question |
| 22.6 | How much time did you usually spend walking on one of those days? | _____ hours per day  _____ minutes per day  Don’t know/Not sure |
| 22.7 | How much time did you spend sitting on a week day? | _____ hours per day  _____ minutes per day  …………Don’t know/Not sure |

1. Ways of spending leisure time: …………………………………………
2. Ever tried to lose weight: Yes/No
3. Food habit: Veg/Non-veg
4. Follow anti-diabetic diet: Yes/No

26.1 frequency of meal per day:……………………

26.2 frequency of eating vegetable per week……………………..

26.3 frequency of eating fruit per week…………………………..

26.4 frequency of eating red meat per week……………………..

26.5 frequency of consuming milk and milk product per week……………………..

26.6 frequency of eating chicken per week………………………….

26.7 frequency of eating eggs per week…………………………….

26.8 frequency of taking fried foods per week……………………..

26.9 frequency of taking sugar and sweet per week………………………….

| **S.N** | **Statement** | Eating/drinking Days per week | Number of serving on those days |
| --- | --- | --- | --- |
| 1 | Frequency of eating fruit |  |  |
| 2 | Frequency of eating vegetable |  |  |
| 3 | Frequency of drinking coffee |  |  |

26.10 dietary compliance reported by physician/endocrinologists

Very good Good Neutral Poor Very Poor

26.11 dietary compliance reported by dietitian

Very good Good Neutral Poor Very Poor

1. Sleep pattern

27.1 Number of nights with difficulty in going to sleep per week………………

- 1. Number of nights with difficulty in maintaining sleep per week…………..

27.3Total number of nights with difficulty in going and maintaining sleep……..

27.8 total numbers hours of sleep per day without difficulty in initiating and maintaining sleep……..

1. **Family history of Illness**

| **Diseases** | **Response** | |
| --- | --- | --- |
| Diabetes in 1^st^ degree relation | Yes (Specify relationship)……………….. | No |
| Hypertension in 1^st^ degree relation | Yes (Specify relationship)……………….. | No |
| Obesity in 1^st^ degree relation | Yes (Specify relationship)……………….. | No |
| Heart Disease in 1^st^ degree relation | Yes (Specify relationship)……………….. | No |

1. **Perceived Level of Stress in last one months**

| **S.N.** | **Statements** | **Never** | **Almost Never** | **Sometimes** | **Fairly Often** | **Very Often** |
| --- | --- | --- | --- | --- | --- | --- |
| 1 | In the last month, how often have you been upset because of something that happened unexpectedly? |  |  |  |  |  |
| 2 | In the last month, how often have you felt that you were unable to control the important things in your life? |  |  |  |  |  |
| 3 | In the last month, how often have you felt nervous and “stressed”? |  |  |  |  |  |
| 4 | In the last month, how often have you felt confident about your ability to handle your personal problems? |  |  |  |  |  |
| 5 | In the last month, how often have you felt that things were going your way? |  |  |  |  |  |
| 6 | In the last month, how often have you found that you could not cope with all the things that you had to do? |  |  |  |  |  |
| 7 | In the last month, how often have you been able to control irritations in your life? |  |  |  |  |  |
| 8 | In the last month, how often have you felt that you were on top of things? |  |  |  |  |  |
| 9 | In the last month, how often have you been angered because of things that were outside of your control? |  |  |  |  |  |
| 10 | In the last month, how often have you felt difficulties were piling up so high that you could not overcome them? |  |  |  |  |  |

1. **Anthropometric Measurement:**

| **Items** | **Measurement Values** |
| --- | --- |
| Height(cm) |  |
| Weight (Kg) |  |
| Waist circumference(cm) |  |
| Blood Pressure(mm of hg)  First Reading (systolic/diastolic)  Second Reading (systolic/diastolic) | ………………………………….  ………………………………….  …………………………………. |
| BMI |  |

1. **Laboratory Investigations**

| **S.N** | **Tests** | **Measurement Value** | **Date of Investigation** |
| --- | --- | --- | --- |
| **1** | **Lipid Profile** |  |  |
|  | HDL(mg/dl) |  |  |
|  | LDL(mg/dl) |  |  |
|  | Triglyceride(mg/dl) |  |  |
|  | Total cholesterol |  |  |
|  | VLDL |  |  |
| **2** | **Fasting plasma glucose (mg/dl)** |  |  |
| **3** | **HbA1c** |  |  |

**Thank You for your Cooperation**
